# Challenges experienced by newly graduated professional nurses during transition to clinical practice in KwaZulu-Natal, South Africa

**DOI:** 10.4102/curationis.v49i1.2907

**Published:** 2026-10-07

**Authors:** Olufunke O. Oyeniyi, Richard M. Rasesemola, Sidwell Matlala, Lerato Matshaka

**Affiliations:** 1 Department of Nursing, Faculty of Health Sciences, University of Johannesburg, Johannesburg, South Africa

**Keywords:** theory-practice gap, newly graduated professional nurses, clinical competence, transition to practice, nursing education

## Abstract

**Background:**

The transition from nursing education to professional practice is a vulnerable period for newly graduated professional nurses. Despite completing accredited programmes, many face challenges transitioning into clinical practice, affecting their competence development, confidence and patient safety.

**Objectives:**

This study explored challenges of professional integration experienced by newly graduated professional nurses during their mandatory community service year in a public hospital in KwaZulu-Natal, South Africa.

**Method:**

A qualitative exploratory design was employed. Seven newly graduated professional nurses undertaking community service participated in individual interviews. Data were analysed using Tesch’s method. Trustworthiness was ensured through credibility, dependability, confirmability and transferability.

**Results:**

Three themes emerged from the data, reflecting barriers to transition into clinical practice. The first theme was *shortage of resources as a barrier to theory-practice integration*, causing burnout, limited supervision and reduced learning opportunities. The second theme was *lack of support from management during transition to practice*, including poor orientation and limited support from managers and colleagues. The third theme was *difficulty in adapting to the professional nursing role*, including low confidence in clinical tasks, unrealistic expectations and communication challenges within the clinical environment.

**Conclusion:**

The study confirms that challenges experienced by newly graduated professional nurses during transition to professional practice relate to systemic, organisational and contextual factors rather than individual deficits.

**Contribution:**

Improved collaboration between education and practice settings, together with structured mentorship and transition programmes, is essential to support newly graduated nurses, strengthen clinical learning environments and promote safe, competent practice.

## Introduction

Clinical competence is a cornerstone of professional nursing practice and a fundamental determinant of patient safety, quality of care and health system performance (Said 2025). Contemporary healthcare systems increasingly require nurses who can integrate technical proficiency with sound clinical judgement, ethical decision-making, effective communication and the ability to apply theoretical knowledge within complex, high-pressure and resource-constrained environments (Connor et al. 2023). As such, clinical competence extends beyond procedural skill to encompass cognitive, relational and ethical dimensions of practice, with direct implications for patient outcomes, service efficiency and public confidence in healthcare delivery (Tura et al. 2024). In contexts characterised by rising patient acuity, escalating disease burdens and persistent workforce and resource constraints, the presence of clinically competent nurses is indispensable to health system resilience and sustainability (Shibiru et al. 2023).

The United States, Australia and the United Kingdom, just like South Africa, have explicitly articulated clinical competence within their regulatory and legislative frameworks (National Council of State Boards of Nursing [NCSBN] 2017; Nursing and Midwifery Board of Australia [NMBA] 2016; Nursing and Midwifery Council [NMC] 2018; South African Nursing Council [SANC] 2005). The *Nursing Act No. 33 of 2005* defines professional competence as the capacity to integrate knowledge, skills, attitudes, judgement and ethical reasoning within a defined scope of practice (SANC 2005). This framing highlights competence as a multidimensional construct, incorporating accountability, critical thinking and professional conduct alongside technical performance (SANC 2005). Despite this regulatory emphasis, there is evidence from literature that points to a disjunction between the intended outcomes of nursing education and the realities of clinical practice, especially during the transition from student to professional nurse (Bvumbwe & Mtshali 2018; Hansen & Zuma 2024; Moremi et al. 2025).

This disjuncture carries consequences for patient safety and health service quality. Baloyi, Ramathuba and Netshisaulu (2024) report increased rates of adverse events during the first year of practice among newly graduated professional nurses, many of which are preventable. International evidence reflects similar patterns. In China, Xie et al. (2021) identified only moderate levels of core clinical competence among newly graduated professional nurses, particularly in assessment, communication and critical thinking. In Kenya, Washira, Mageto and Mapesa (2017) reported inadequacies in clinical judgement during internship, especially in decision-making and care prioritisation. Locally, Nkgapele (2025) found that newly graduated professional nurses in Limpopo province experienced difficulties with independence and professional commitment during community service, contributing to workplace tension and dissatisfaction among senior staff.

Newly graduated professional nurses constitute a strategically important segment of the nursing workforce (Sharplin, Clarke & Eckert 2025). They enter practice with current theoretical knowledge, emerging technological competencies and potential to contribute to long-term workforce sustainability (Mamabolo & Fombad 2023). However, the transition from education to practice is widely recognised as a critical and vulnerable phase of professional development. During this period, newly graduated professional nurses are confronted with heightened responsibility, complex clinical decision-making demands, unfamiliar organisational contexts and significant emotional and cognitive strain (Jenkins, Carter & Smythe 2025). Matlhaba, Pienaar and Sehularo (2021) characterise this transition as a period requiring deliberate, structured support, noting that insufficient supervision and mentorship can transform early professional practice into a source of distress rather than consolidation and growth.

In South Africa, this transition is formally mediated through a mandatory year of community service. In terms of the *Nursing Act No. 33 of 2005*, newly graduated professional nurses in general, psychiatric, community nursing and midwifery must complete community service prior to full professional registration (SANC 2005). This policy mechanism is intended to consolidate clinical competence, support professional socialisation and expose graduates to diverse healthcare settings. Zulu et al. (2025) highlight the pivotal role of this period in shaping clinical skill development, professional identity formation and longer-term career trajectories. However, accumulating evidence suggests that community service frequently falls short of these policy objectives (Zambodla, Williams & Ricks 2022).

Rather than functioning as a structured period of supported consolidation, community service often places newly graduated professional nurses in clinical environments characterised by high workloads, staffing shortages, inconsistent practices and limited supervisory capacity (Mabusela & Ramukumba 2021; Matlhaba et al. 2021). In many under-resourced public healthcare settings, community service nurses are expected to assume autonomous clinical responsibilities soon after placement, frequently in the absence of adequate orientation, mentorship or protected learning opportunities (Khunou 2023). This premature assumption of full professional responsibility intensifies the theory-practice gap, undermines confidence and increases vulnerability to stress, moral distress and burnout, revealing a sustained disconnect between policy intent and implementation realities (Hansen & Zuma 2024).

Although nursing education programmes aim to equip graduates with robust theoretical foundations and essential clinical skills, many newly graduated professional nurses report significant difficulty translating this knowledge into effective practice (Almarwani 2025). Complex patient presentations, chronic resource limitations, high patient-to-nurse ratios and fragmented support structures expose misalignments between academic preparation and workplace expectations (Malematja, Nkosi & Nene 2025). Note that newly graduated professional nurses frequently struggle with clinical assessment, symptom management, multidisciplinary communication, care prioritisation and reflective clinical reasoning, competencies that are central to safe and effective practice (Matlhaba 2025).

Against this backdrop, there is a pressing need for empirical evidence that elucidates the challenges faced by newly graduated professional nurses during the transition into clinical practice. The present study, therefore, explores challenges related to transitioning to clinical practice among newly graduated professional nurses in a public hospital in KwaZulu-Natal, with the aim of informing more effective, contextually responsive transition support mechanisms capable of strengthening clinical competence, safeguarding patient care and enhancing workforce sustainability.

Despite completing accredited nursing programmes and meeting regulatory requirements, many newly graduated professional nurses experience substantial difficulty transitioning into clinical practice during community service. National and international evidence indicates persistent deficits in clinical competence, confidence and decision-making, exacerbated by resource constraints, limited supervision, inadequate mentorship and unrealistic role expectations. Within South Africa’s resource-constrained public health system, these challenges undermine the intended developmental function of community service and highlight a critical gap between regulatory expectations and practice realities, indicating the need to examine and elucidate structural, educational and workplace challenges faced by the newly graduated professional nurses during their transitional phase. Therefore, the purpose of the study was to explore and describe challenges experienced by newly graduated professional nurses during their transition to clinical practice in a public hospital.

## Research methods and design

### Study design

A qualitative, exploratory, descriptive and contextual research design was employed to gain an in-depth understanding of the challenges of newly graduated professional nurses during their community service year. This design was considered appropriate because of the complexity, context-bound and individual perceptions and lived experiences related to the challenges experienced by the newly graduated professional nurses during transition to practice. The qualitative approach enabled the researcher to capture rich, detailed narratives that reflected participants’ subjective interpretations of their challenges within the clinical environment as newly graduated professional nurses. An exploratory design was used because limited empirical evidence exists regarding the specific challenges among newly graduated professional nurses in public hospitals in KwaZulu-Natal. This approach allowed the researcher to investigate the phenomenon without preconceived assumptions and to identify emerging patterns directly from participants’ accounts. The descriptive component of the design facilitated a comprehensive portrayal of participants’ experiences, perceptions, and challenges as they naturally occurred in practice. By describing these experiences in detail, the study provided insight into how clinical competence is shaped during the community service period. The contextual nature of the design ensured that findings were interpreted within the specific organisational, cultural, and resource-related realities of a public hospital setting.

### Setting

The study was conducted at a public district hospital located in the KwaZulu-Natal province of South Africa. The hospital functions as a Level 1 district healthcare facility providing 24-h services to a predominantly rural population. It offers a wide range of healthcare services, including medical, surgical, maternity, paediatric, emergency and outpatient care, and serves as a primary placement site for newly graduated professional nurses completing their compulsory community service. This setting was purposively selected because of its relevance to the study objectives. As a public hospital, the facility reflects the realities of the South African public healthcare system, including resource constraints, staff shortages, high patient loads and diverse patient needs. Newly graduated professional nurses in this setting are frequently exposed to complex clinical situations and are often expected to assume professional responsibilities early in their placement. Conducting the study in this environment provided an ideal context for challenges during the transition from student to professional nurse. Data collection took place within the hospital, allowing participants to reflect on their experiences in their natural work environment.

### Population and sampling

The study population comprised newly graduated professional nurses who were undertaking their first year of community service at the selected public hospital. These nurses had successfully completed an accredited nursing degree and were registered with the SANC in the community service category. This group was considered information-rich, as participants were directly experiencing the transition from nursing education to independent clinical practice. Purposive sampling was used to select participants who met the inclusion criteria and were able to provide relevant and meaningful insights into the phenomenon under study. Inclusion criteria required that participants be newly graduated professional nurses currently in their community service year, employed at the selected hospital, and willing to participate voluntarily in the study. Nurses who had already completed their community service year or who were not allocated to the hospital during the data collection period were excluded.

Seven in-depth interviews were conducted. Data collection and analysis occurred concurrently, and thematic saturation was reached at the seventh interview, when iterative coding and constant comparison revealed no new themes, categories or substantive insights. Saturation was evidenced by repetition of patterns across participants and conceptual completeness of the emerging findings, indicating adequate coverage of the phenomenon (Guest, Bunce & Johnson 2006; Saunders et al. 2018). The final sample size was therefore considered sufficient. The use of a small, purposively selected sample was methodologically appropriate for this qualitative study, which aimed to explore complex, subjective experiences rather than achieve statistical generalisation (Crouch & McKenzie 2006; Patton 2015). Consistent with qualitative research traditions, the sample size prioritised analytic depth, contextual richness and detailed engagement with participants’ narratives, thereby enhancing the credibility and trustworthiness of the findings (Creswell & Poth 2018).

### Data collection

Data were collected through unstructured, in-depth individual interviews. This method allowed participants to express their views freely and in detail, thereby capturing rich descriptive data related to their transition from nursing education to clinical practice. Interviews were conducted online using the Zoom^®^, which enabled flexibility owing to shift work and ensured privacy and confidentiality of participants. All interviews were conducted in English and were audio-recorded with the participants’ written informed consent. Each interview lasted approximately 45 min – 60 min. The central open-ended question guiding the interviews was: ‘*How has it been for you as a newly graduated professional nurse?*’ allowing participants to reflect broadly on their experiences since entering professional practice. Probing questions were used to elicit further information. Field notes were recorded during and immediately after interviews to capture contextual observations and support data interpretation. Data collection continued until data saturation was achieved, when no new information emerged from the interviews.

### Data analysis

Data were analysed using Tesch’s eight-step method of qualitative data analysis (Tesch 1990). Audio-recorded interviews were transcribed verbatim by the researcher shortly after data collection to ensure accuracy and familiarity with the data. The researcher read and re-read the transcripts to gain an overall understanding of the content before commencing coding. Open coding was used to identify significant statements and meaning units related to participants’ experiences. Similar codes were grouped into categories, which were further refined and organised into themes and subthemes reflecting patterns across the data. Constant comparison was applied throughout the analysis process to identify similarities and differences within and across participants’ narratives. An independent coder experienced in qualitative research analysed the transcripts independently to enhance rigour and minimise researcher bias (Lincoln & Guba 1985). Consensus meetings were held between the researcher and the independent coder to discuss and agree on the final themes and subthemes. This process strengthened confirmability and ensured that the findings accurately reflected participants’ experiences.

### Trustworthiness

Trustworthiness of the study was ensured using the criteria of credibility, dependability, confirmability and transferability, as described by Lincoln and Guba (1985) and Creswell and Poth (2018). Credibility was established through prolonged engagement with the participants and the research context, which allowed the researcher to develop rapport and gain an in-depth understanding of participants’ experiences. Member checking was conducted informally by clarifying meanings during interviews and verifying interpretations to ensure that the data accurately reflected participants’ perspectives. Dependability was ensured through a detailed description of the research design, data collection, and data analysis processes, which allowed for the study to be audited. An audit trail was maintained, including verbatim transcripts, field notes and reflective notes, to demonstrate consistency and transparency in the research process. Confirmability was achieved by minimising researcher bias using an independent coder who analysed the data separately. Consensus discussions between the researcher and the independent coder were held to agree on the final themes and subthemes. Documentation of decisions made during data analysis supported the objectivity of the findings. Transferability was supported through rich, thick descriptions of the research context, participants and study procedures, enabling readers to assess the applicability of the findings to similar settings.

### Ethical considerations

Ethical clearance was obtained from the Faculty of Health Sciences Research Ethics Committee (REC) at the University of Johannesburg (REC-1661-2022). Permission to conduct the study was granted by the KwaZulu-Natal Department of Health and hospital management. Participation was voluntary, and written informed consent was obtained from all participants. Confidentiality and anonymity were maintained through the use of codes.

## Results

Participants’ demographic information is presented in Table 1.

**TABLE 1:** Demographic information.

Participant number	Age (years)	Gender	Educational qualification
1	25	Male	Baccalaureus Technologiae: Nursing Science
2	25	Female	Baccalaureus Curationis: Nursing
3	24	Female	Baccalaureus Curationis: Nursing
4	26	Female	Baccalaureus Technologiae: Nursing Science
5	24	Female	Baccalaureus Curationis: Nursing
6	24	Female	Baccalaureus Technologiae: Nursing Science
7	24	Female	Baccalaureus Curationis: Nursing

Table 1 presents the demographic and educational characteristics of the seven participants included in the study. The sample comprised both male and female newly graduated professional nurses employed as community service nurses in a public hospital in KwaZulu-Natal. Participants were relatively homogenous in age, ranging from 24 years to 26 years.

In terms of educational preparation, participants held either the *Baccalaureus Curationis* (BCur) or the *Baccalaureus Technologiae* (BTech) in Nursing Science. Although these qualifications are offered by different nursing education institutions, with the BCur traditionally offered at conventional universities and the BTech historically offered at universities of technology, all programmes are regulated by SANC under Regulation R425. Both qualifications require a minimum duration of 4 years, meet the same professional and clinical requirements, and lead to registration as a Professional Nurse and Midwife. Importantly, the articulation pathways for both qualifications are comparable, allowing graduates access to similar postgraduate opportunities.

### Themes and subthemes

Three themes and seven supporting subthemes emerged from the analysis as presented in Table 2.

**TABLE 2:** Themes and subthemes.

Themes	Subthemes
1. Shortage of resources as a barrier to theory-practice integration	1.1 Professional nurse shortages leading to burnout and reduced supervision 1.2 Limited learning opportunities because of shortages of hospital equipment
2. Lack of support from management during transition to practice	2.1 Inadequate management support and absence of structured orientation 2.2 Insufficient support from professional nurses and doctors
3. Difficulty in adapting to the professional nursing role	3.1 Lack of confidence in performing ward activities as newly qualified professional nurses 3.2 Unrealistic role expectations based on academic qualifications 3.3 Language and communication barriers in clinical practice

### Theme 1: Shortage of resources as a barrier to theory-practice integration

This theme reflects how inadequate human and material resources hinder newly graduated professional nurses’ ability to consolidate theoretical knowledge into safe clinical practice.

#### Subtheme 1.1: Professional nurse shortages leading to burnout and reduced supervision

Shortages of professional nurses had a profound impact on newly graduated professional nurses’ transition into clinical practice. Staffing constraints resulted in increased workloads and emotional exhaustion, as newly graduated professional nurses were frequently required to assume responsibilities beyond their level of experience. The absence of adequate staffing also reduced opportunities for supervision, mentorship and guided learning, leaving new graduates to practise with minimal support. As a result, newly graduated professional nurses were compelled to function independently before achieving full clinical readiness, intensifying feelings of anxiety, self-doubt and role strain. These conditions further widened the gap between theoretical preparation and clinical expectations, undermining the effective integration of theory into practice during the critical transition period. Consequently, professional nurse shortages emerged as a significant barrier to bridging the theory-practice divide in the early stages of professional nursing practice. One participant illustrated how staff shortages resulted in inappropriate placement of newly graduated professional nurses without adequate preparation, limiting supervision and increasing role strain:

‘There is a shortage of staff, so since I am a newly graduate, they are trying to place me wherever. So, these things affect me a lot because if they take me to psychiatry ward and I don’t know … you find out in psychiatry ward, it’s only a Professional Nurse and Enrolled Nurses and they will call me and say, there is no one in psychiatry ward, go and work there.’ (Participant 1, Male, 25 years)

Another participant highlighted the direct impact of professional nurse shortages on the availability of supervision, forcing newly graduated professional nurses to practise independently despite their transitional status:

‘Isn’t it for us as community services, we still need guidance. But sometimes, you end up not getting that much guidance because there’s shortage of staff. There’s no one else who would actually give us guidance, you end up doing everything alone without guidance.’ (Participant 2, Female, 25 years)

Quotations further reflected participants’ perception that workforce shortages led to premature independence:

‘The lack of staff is what is making them to push community services to work alone, of which it’s not right.’ (Participant 5, Female, 24 years)

Another participant reflected on how staffing shortages forced newly graduated professional nurses to make compromises in care delivery:

‘Sometimes, you have to prioritise what to do and what not to do because there is no staff, which is not good for the patient and not good for our competency as well.’ (Participant 3, Female, 24 years)

#### Subtheme 1.2: Limited learning opportunities because of shortages of hospital equipment

Participants’ accounts indicate that inadequate and non-functional hospital equipment significantly limited learning opportunities during their transition to clinical practice. The absence of essential diagnostic and monitoring equipment curtailed newly graduated professional nurses’ ability to practise clinical skills and translate theoretical knowledge into real patient care situations. As a result, routine and advanced procedures were either delayed, modified or omitted altogether, reducing exposure to critical learning experiences. These challenges not only constrained hands-on clinical learning but also compromised diagnostic and treatment processes, reinforcing discrepancies between what was taught in nursing education and what was feasible in practice. Consequently, shortages of hospital equipment emerged as a key factor perpetuating the theory-practice gap and hindering the development of clinical competence during the community service period. One participant illustrated how the absence of basic equipment limited the ability to perform routine clinical procedures, thereby restricting opportunities to practise and consolidate theoretical knowledge:

‘Like in post-natal, we are supposed to weigh a baby daily. We don’t have a weighing scale. I’ll have to run to special care, and while running that side, if there’s an emergency for a patient, who’s going to attend to that? So, we’re missing a lot of things.’ (Participant 3, Female, 24 years)

Another participant also demonstrated how equipment shortages compromised diagnostic processes and reduced exposure to learning opportunities associated with patient assessment and clinical decision-making:

‘There was a point where we had no X-ray machine. So, it was hard for the doctor to make a diagnosis for the patient, to prescribe correct treatment. Sometimes the patient will stay for a week without an X-ray, delaying healing and our learning as well.’ (Participant 3, Female, 24 years)

Participants further highlighted how non-functional equipment limited newly graduated professional nurses’ ability to apply theoretical knowledge to obstetric practice:

‘The CTG [*cardiotocography*] machine is there but it doesn’t work properly. Sometimes it has no probes, so you can’t monitor contractions and foetal heartbeat properly. It becomes difficult to manage the patient.’ (Participant 7, Female, 24 years)

Quotation from one participant explicitly reflected the theory-practice gap created by inadequate equipment and resources in the clinical setting:

‘Because of lack of resources, there are things you never get to do. Like abnormal deliveries, you only learn them in theory, but you never practise them in the ward.’ (Participant 7, Female, 24 years)

### Theme 2: Lack of support from management during transition to practice

This theme highlights how insufficient organisational and interpersonal support negatively affected the transition from nursing education to clinical practice.

#### Subtheme 2.1: Inadequate management support and absence of structured orientation

Participants described receiving minimal or no formal orientation upon placement, which left them inadequately prepared for unit-specific procedures, policies and role expectations. The absence of structured orientation and consistent management support contributed to heightened anxiety and uncertainty during the early stages of clinical practice. Newly graduated professional nurses were often expected to assume responsibilities without clear guidance, compelling them to rely on trial-and-error learning while delivering patient care. This lack of support limited opportunities for structured supervision and guided application of theoretical knowledge, thereby impeding the effective integration of theory into practice. Consequently, inadequate management support and the absence of formal orientation intensified the theory-practice gap and posed significant challenges to competence development during the transition to professional practice.

Participants highlighted the lack of a structured and comprehensive orientation programme, leaving newly graduated professional nurses unclear about expectations, supervision and role boundaries:

‘It was just plain introduction to the hospital, like plain orientation. “This is the ward that you’ll be working in.” They didn’t specify if community service nurses will be paired with a registered nurse or will be working alone.’ (Participant 5, Female, 24 years)

Participants further illustrated how inadequate management-led orientation resulted in uncertainty and forced newly graduated nurses to learn informally while delivering patient care:

‘Most of the time, there was no proper orientation. You are just allocated to the ward and expected to start working immediately, even when you don’t know how things are done.’ (Participant 3, Female, 24 years)

‘Sometimes you are left in the ward alone, and you are still a community service nurse. There is no one explaining policies or how things are done in that ward.’ (Participant 5, Female, 24 years)

Participants also reflected on the absence of managerial and supervisory support during transition, with unrealistic expectations replacing structured guidance:

‘So, when you get here, instead of them helping you with some of the things, they just assume because you went to university you know everything. When you make mistakes, they ask you “Where did you go to school?”’ (Participant 7, Female, 24 years)

#### Subtheme 2.2: Insufficient support from professional nurses and doctors

Participants’ accounts revealed that limited supervision, negative attitudes and unrealistic expectations from professional nurses and doctors significantly hindered learning and confidence development among newly graduated professional nurses. Rather than experiencing mentorship and guided support during their transition, participants encountered criticism, comparison with more experienced staff and assumptions that academic qualifications equated to full clinical competence. These interactions undermined psychological safety and discouraged help-seeking behaviours, leaving newly graduated professional nurses reluctant to ask questions or clarify uncertainties. The absence of consistent professional support restricted opportunities for guided learning and reflective practice, forcing new graduates to navigate complex clinical situations independently. One participant reflected on the lack of emotional and professional support from senior nurses, with comparison and criticism replacing guidance and mentorship:

‘None of them are willing to help. They will even compare you to the enrolled nurses, saying they know better than you. One mistake, they say, “We told you, you don’t know anything.”’ (Participant 5, Female, 24 years)

Another one reflected on unrealistic expectations and dismissive attitudes from professional nurses and doctors, limiting opportunities for learning and clarification:

‘Instead of helping you, they give you attitude. They expect that because you went to university you must know everything.’ (Participant 7, Female, 24 years)

Participants further highlighted the emotional toll of inadequate collegial support, demonstrating how lack of assistance negatively affected confidence, motivation, and well-being:

‘I was struggling to the point that when I wake up in the morning, I feel like, “Can I go to work?” because the sisters are not willing to help you and they make fun of you.’ (Participant 4, Female, 26 years)

Another participant indicated the presence of gaps in professional supervision, indicating insufficient support from appropriately qualified:

‘I ended up being supervised by an enrolled nursing assistant instead of a registered nurse or operational manager.’ (Participant 3, Female, 24 years)

### Theme 3: Difficulty in adapting to the professional nursing role

This theme captures the challenges newly graduated professional nurses experienced while adjusting from student roles to independent professional practice.

#### Subtheme 3.1: Lack of confidence in performing ward activities as newly qualified professional nurses

Participants reported heightened anxiety and self-doubt when expected to perform clinical tasks independently, largely attributable to limited practical exposure during training and insufficient support during the community service period. Their accounts further indicate that lack of confidence among newly graduated professional nurses during the transition to practice was shaped by inadequate experiential learning opportunities and unrealistic expectations from senior staff, which hindered independent performance of ward activities, intensified anxiety when applying theoretical knowledge and ultimately reinforced the theory-practice gap during early clinical practice. Participants reflected how a lack of confidence undermined participants’ ability to function independently in ward activities, despite expectations of competence:

‘I think competency also comes from you being confident, right? But I am not confident and cannot even work on my own. Sometimes the sisters feel like because you are from a four-year course you must just do the work.’ (Participant 4, Female, 26 years)

Participants’ perceptions were that insufficient experience contributed directly to their lack of confidence in performing ward activities:

‘I’m a newly graduate and remember, nursing goes hand in hand with experience. So, if I don’t have experience, there is no way I can know more things or different approaches to nursing.’ (Participant 4, Female, 26 years)

‘A lack of exposure and experience. The experienced sisters expect us as newly graduates to know more than we know. They assume that because we are graduates, we know everything.’ (Participant 5, Female, 24 years)

Other participants reflected on how fear of judgement and unrealistic expectations further diminished confidence in carrying out clinical duties:

‘I don’t know some of the things, I’m afraid to even ask because they expect you to know everything. That affects one’s confidence and makes one afraid to work alone.’ (Participant 5, Female, 24 years)

#### Subtheme 3.2: Unrealistic role expectations based on academic qualifications

Participants’ accounts indicate that unrealistic expectations rooted in academic qualifications fostered assumptions of immediate clinical competence. The necessity for experience-based learning and supervision was overlooked, which further heightened anxiety and reduced confidence. Questioning and mentorship interactions were discouraged and ultimately widened the theory-practice gap during the transition from nursing education to professional clinical practice. The following quotation from participant demonstrates how academic qualification was equated with full clinical competence, resulting in unrealistic expectations of independent practice:

‘Everyone expects me to know everything just because I have graduated. Since I’m a community service nurse, I’m still supposed to work under supervision, but they act like I must already know everything.’ (Participant 1, Male, 25 years)

Another participant further highlighted the disconnect between academic preparation and experiential learning, reinforcing unrealistic assumptions about readiness for practice:

‘They say you went to university for four years, so you must know everything. But experience is different from theory.’ (Participant 7, Female, 24 years)

Others highlighted how unrealistic expectations, and comparisons with experienced nurses discouraged help-seeking behaviours, heightened pressure and contributed to feelings of inadequacy, thus undermining their confidence during the transition period:

‘Some of the things, you won’t know, but you are even afraid to ask because they expect you to know everything. That affects your confidence.’ (Participant 5, Female, 24 years)

‘They compare you with nurses who have been working for many years, forgetting that you are fresh from school, even though you have a degree.’ (Participant 4, Female, 26 years)

#### Subtheme 3.3: Language and communication barriers in clinical practice

Participants’ narratives revealed that language and communication barriers posed a significant challenge during their transition to clinical practice. Newly graduated professional nurses described difficulties adapting to clinical environments where patients and colleagues predominantly communicated in languages different from their own. These challenges heightened anxiety, impeded effective nurse-patient interactions, and complicated the delivery of timely and appropriate care. Limited language proficiency further undermined confidence when explaining procedures, assessing patients and participating in multidisciplinary communication, often necessitating reliance on others for translation. As a result, language and communication barriers restricted the effective application of theoretical knowledge in practice and contributed to feelings of professional inadequacy during the early stages of professional nursing practice.

Participants reflected on how language differences heightened anxiety and made adaptation to clinical practice more challenging:

‘The new environment, the anxiety. Now you are learning new people who speak a different language from you. You have to adapt, and sometimes it’s difficult to learn the language in a day.’ (Participant 6, Female, 24 years)

Participants further reflected on the struggle to communicate effectively with patients and colleagues in an unfamiliar language, affecting confidence and daily clinical functioning:

‘I’m trying my best to survive here because I’m not [*isi*] Zulu, so I have to adapt to their language. To be honest, it’s not easy.’ (Participant 1, Male, 25 years)

Other participants highlighted how language barriers interfered with effective communication, timely care delivery and clinical decision-making:

‘Sometimes patients explain their problems in a language you don’t fully understand, and you have to ask someone else to translate, which delays your care and makes you feel less confident.’ (Participant 5, Female, 24 years)

‘Even explaining procedures becomes difficult when you are not fluent in the patient’s language, and that makes you doubt yourself as a nurse.’ (Participant 4, Female, 26 years)

## Discussion

The findings of this study highlight the complex factors shaping challenges faced by the newly graduated professional nurses during the transition to practice. The findings reveal that resource constraints, including shortages of staff and equipment, function as structural barriers that hinder effective theory-practice integration. In addition, insufficient organisational and interpersonal support, absence of structured orientation and limited supervision exacerbate transition challenges. Participants further reported difficulties adapting to the professional nursing role, compounded by reduced confidence and unrealistic expectations linked to academic qualifications rather than experiential readiness. Language and communication barriers within clinical environments emerged as an added challenge, undermining effective care delivery and learning.

### Shortage of resources as a barrier to theory-practice integration

The findings highlight that deficits in both human and material resources pose significant barriers to effective theory-practice integration among newly graduated professional nurses. Staffing shortages required nurses to assume full clinical responsibilities prematurely, often in the absence of adequate supervision and mentorship, thereby limiting structured learning opportunities and contributing to emotional fatigue. Simultaneously, shortages of essential and functional equipment curtailed hands-on clinical exposure, constrained skills acquisition and intensified the disconnect between theoretical preparation and clinical practice. These findings are consistent with existing literature that identifies inadequate staffing and resource constraints as major impediments to professional development and contributors to psychological distress among nurses (Kim & Kim 2021; Madolo & Hloba 2023; Mafumo & Maputle 2025; Oshodi & Sookhoo 2024; Zondi & Mkhize 2025). Therefore, addressing staffing shortfalls is critical to ensuring adequate supervision, mentorship, and protected learning time during the early stages of professional practice, while sustained investment in functional clinical equipment is essential to support competencies development and ensure safe patient care (Alhassan et al. 2025; Mlaba & Emmamally 2019). Failure to address these structural constraints risks undermining clinical competence, compromising patient safety, and exacerbating nurse attrition, with significant implications for workforce sustainability and healthcare quality (Rauf et al. 2024).

### Lack of support from management during transition to practice

The absence of structured orientation and limited management support contributed to heightened anxiety and uncertainty among newly graduated professional nurses. Participants frequently relied on trial-and-error learning, reflecting weak organisational transition mechanisms. Inadequate collegial support from professional nurses and medical staff further eroded confidence, discouraged help-seeking behaviours and constrained opportunities for mentored learning. These findings highlight significant gaps in organisational responsibility for supporting early professional transition and the need for formalised, standardised transition-to-practice programmes. Consistent with previous research, well-designed orientation and transition-to-practice programmes play a critical role in facilitating the transition from academic preparation to clinical practice by providing structured mentorship and preceptorship, reducing reality shock, promoting safe practice and improving retention (Aldosari, Pryjmachuk & Cooke 2021; Rush et al. 2013). Conversely, poorly structured or inadequately implemented orientation initiatives that are misaligned with clinical demands have limited impact on nurse performance and may exacerbate transition shock. This evidence demonstrates that the effectiveness of transitional support depends not merely on its availability but on its structure, comprehensiveness, and organisational commitment. Management-supported, clinically relevant and adequately resourced orientation and transition-to-practice systems are therefore essential to enhance professional competence, reduce early-career distress and mitigate nurse turnover.

Furthermore, healthcare organisations must implement clear orientation guidelines, transitional supervision models and robust accountability structures to optimise support for newly qualified professional nurses. Strengthening professional and interprofessional collaboration fosters psychological safety, enhances learning, and builds clinical confidence during the transition period. Studies by Zonneveld, Conroy and Lines (2025); Vostrý et al. (2024), and Höglander et al. (2025) consistently emphasise that clearly articulated orientation frameworks, structured transition activities and effective supervision are central to successful professional integration. Well-designed transition systems have been shown to improve preparedness, efficiency and self-confidence, while their absence is linked to decreased performance and increased turnover risk. Importantly, these findings further underscore the value of accountability mechanisms and validated transition guidelines, highlighting the need for strengthened collaboration between educational institutions and clinical environments to ensure alignment between academic learning and practice expectations. This evidence suggests that effective transition into professional practice requires an integrated, system-level approach that combines structured orientation, sustained supervision, and inter-institutional collaboration to support competence development, workforce stability and safe patient care.

### Difficulty in adapting to the professional nursing role

Participants struggled to adapt to the professional nursing role as a result of limited experiential learning, unrealistic expectations of competence based on academic qualifications and fear of judgement. These factors collectively diminished confidence in performing ward activities and widened the theory-practice gap during early practice. Chen et al. (2024) have similar findings: a significant ‘mismatch’ between workplace expectations and the clinical realities faced by novice nurses was identified. They further highlighted that new graduates often feel unprepared and insecure as a result of a lack of hands-on experience, which leads to increased anxiety and a sense of inadequacy when performing clinical skills independently. Furthermore, Gautam et al. (2023) also reported on unrealistic expectations from senior staff and a fear of making mistakes (judgement) by newly graduated professional nurses, which left them fearful and bereft of confidence. The study concludes that inadequate mentorship and poor clinical immersion during training directly contribute to these transitional struggles. The difficulties adapting to the professional nursing role suggest a need for gradual role assumption and competency-based transition pathways. As suggested by Chipu (2025) and Lindblom et al. (2025), supportive supervision and reflective practice opportunities are essential to build confidence and reduce anxiety. Without such mechanisms, newly graduated professional nurses may experience prolonged adjustment difficulties, affecting performance and well-being.

Academic qualifications were frequently assumed to equate to full clinical competence, with insufficient recognition of the need for experience-based learning and sustained supervision. This misalignment placed considerable pressure on newly graduated professional nurses, discouraged questioning and constrained competence development during the community service period. Similar concerns have been reported in the South African context, where unrealistic expectations of clinical readiness have been shown to negatively affect newly graduated professional nurses’ confidence and professional development (Matlhaba et al. 2021). Findings from this study further suggest that undergraduate nursing programmes often provide limited preparation for the complexity of real-world clinical roles. Inadequate leadership and minimal support from departmental managers and educators leave graduates to navigate professional responsibilities with insufficient supervision, thereby undermining their transition into practice.

The persistent assumption that academic qualification alone ensures clinical competence indicates the need for greater awareness among nurses, medical practitioners and managers regarding the developmental nature of transitional competence (Charette et al. 2023; Doğan & Tarhan 2026). Newly graduated professional nurses require protected learning environments that prioritise supervision, feedback, and gradual responsibility allocation. Clear communication regarding the scope of practice, learning needs, and support requirements of community service nurses is, therefore, essential to align expectations, foster mentorship and safeguard experiential learning opportunities. Addressing these issues is critical to strengthening competence development, enhancing professional confidence and supporting safe and sustainable nursing practice.

Language differences between newly graduated professional nurses, patients, and colleagues compounded transition challenges by hindering effective communication, delaying care and undermining confidence. These barriers further restricted the application of theoretical knowledge in practice. Language and communication challenges highlight the importance of culturally and linguistically responsive practice environments. This study highlights that newly graduated professional nurses in South Africa’s Community Service Programme often struggle when placed in rural clinics where they are sometimes the most senior staff. It explicitly notes that language differences between practitioners and patients act as a significant barrier, leading to communication breakdowns and feelings of inadequacy among the nurses.

Healthcare institutions should support basic clinical language training, encourage team-based communication strategies and promote inclusive practices to mitigate anxiety and ensure effective nurse-patient interactions (Hlosana-Lunyawo & Yako 2013). This study identifies that poor interpersonal skills and communication failures are major obstacles in the transition from student to professional nurse. It argues that without a culturally and linguistically responsive environment, new graduates cannot effectively apply their theoretical knowledge, resulting in ‘missed care’ and compromised patient safety (Doğan & Tarhan 2026).

The literature indicates that effective management of language and communication barriers in healthcare requires an integrated, system-level approach that combines targeted training, organisational support and curriculum reform. Key recommendations include the provision of ongoing cultural competence and language training to enhance linguistic sensitivity and local language proficiency among healthcare workers (Ali & Johnson 2017; Falatah, Al-Harbi & Alhalal 2022), alongside structural support such as access to professional interpreter services and linguistically responsive workplace cultures that normalise help-seeking (Ali & Johnson 2017; Doğan & Tarhan 2026). Improved preparation for community service through pre-placement orientation and structured mentorship, particularly in rural settings, has been shown to reduce reality shock and strengthen communication capacity among newly graduated professional nurses (Hlosana-Lunyawo & Yako 2013). In parallel, strengthening undergraduate curricula through focused development of interpersonal communication skills and the use of simulated multilingual clinical scenarios can better prepare graduates for language discordance in practice (Eklicks et al. 2024). These strategies support safer patient care, enhance professional confidence and facilitate more effective transition into practice within linguistically diverse healthcare contexts.

These findings demonstrate that the challenges for the newly graduated professional nurses are sustained by an interplay of systemic constraints, inadequate organisational support and contextual challenges encountered during the transition from nursing education to professional practice. These results confirm that effective transition and professional integration cannot be achieved through educational preparation alone but require coordinated and sustained collaboration between nursing education institutions, healthcare organisations and regulatory bodies.

### Strengths and limitations

This study demonstrates that transition challenges experienced by newly graduated professional nurses arise from systemic shortcomings rather than individual inadequacy. Staffing and resource constraints, weak orientation structures, unrealistic expectations of competence, limited mentorship and language discordance collectively widen the theory-practice gap and promote premature role assumption. The findings indicate the need for a coordinated, system-level transition framework incorporating structured orientation, sustained supervision, gradual responsibility allocation, and management support. However, the single-site design and small sample size limit generalisability.

### Recommendations

The first recommendation relates to strengthening clinical exposure and hands-on practice within nursing education to narrow the theory-practice gap identified in this study. Undergraduate programmes should prioritise experiential learning through increased supervised clinical placements, skills laboratories and simulation that reflects real-world resource, workload and communication challenges. Early and sustained clinical immersion can support competence development, reduce anxiety and better prepare graduates for the realities of professional practice.

Additionally, implementing structured orientation, mentorship and transition-to-practice programmes is critical to support newly graduated professional nurses during early professional transition. Standardised, well-resourced transition frameworks that include protected learning time, sustained supervision and gradual role assumption can mitigate reality and transition shock. Management support for mentorship and preceptorship is essential to promote confidence, competence consolidation and retention.

Lastly, enhancing collaboration between educational institutions and healthcare facilities is necessary to ensure alignment between academic preparation and clinical expectations. Shared responsibility for transition planning, clear communication about graduate scope and learning needs and co-developed transition guidelines can strengthen continuity from education to practice. Such collaboration supports realistic competency expectations, improves learning environments and contributes to safer, more sustainable workforce integration.

## Conclusion

This study demonstrates that the transition challenges faced by newly graduated professional nurses are rooted in system-level deficiencies rather than individual shortcomings. Persistent staffing and resource constraints, fragmented orientation and transition structures, unrealistic expectations of clinical competence, limited supervision and contextual communication challenges collectively sustain the theory-practice gap and undermine competence development. The findings highlight that transitional competence is developmental and contingent on supportive clinical environments. Effective transition to professional practice therefore requires coordinated, well-resourced and management-supported transition-to-practice frameworks, grounded in collaboration between education and service sectors. Addressing these structural determinants is essential to safeguarding patient safety, strengthening workforce sustainability and enabling confident, competent nursing practice.
